# Native mass spectrometry identifies the HybG chaperone as carrier of the Fe(CN)_2_CO group during maturation of *E. coli* [NiFe]-hydrogenase 2

**DOI:** 10.1038/s41598-021-03900-w

**Published:** 2021-12-21

**Authors:** Christian Arlt, Kerstin Nutschan, Alexander Haase, Christian Ihling, Dirk Tänzler, Andrea Sinz, R. Gary Sawers

**Affiliations:** 1 Institute of Pharmacy, Center for Structural Mass Spectrometry, Martin-Luther University Halle-Wittenberg, Kurt-Mothes-Str. 3a, 06120 Halle (Saale), Germany; 2 Institute for Biology/Microbiology, Martin-Luther University Halle-Wittenberg, Kurt-Mothes-Str. 3, 06120 Halle (Saale), Germany

**Keywords:** Metalloproteins, Mass spectrometry, Metals, Iron, Multienzyme complexes, Biochemistry, Enzymes, Oxidoreductases

## Abstract

[NiFe]-hydrogenases activate dihydrogen. Like all [NiFe]-hydrogenases, hydrogenase 2 of *Escherichia coli* has a bimetallic NiFe(CN)_2_CO cofactor in its catalytic subunit. Biosynthesis of the Fe(CN)_2_CO group of the [NiFe]-cofactor occurs on a distinct scaffold complex comprising the HybG and HypD accessory proteins. HybG is a member of the HypC-family of chaperones that confers specificity towards immature hydrogenase catalytic subunits during transfer of the Fe(CN)_2_CO group. Using native mass spectrometry of an anaerobically isolated HybG–HypD complex we show that HybG carries the Fe(CN)_2_CO group. Our results also reveal that only HybG, but not HypD, interacts with the apo-form of the catalytic subunit. Finally, HybG was shown to have two distinct, and apparently CO_2_-related, covalent modifications that depended on the presence of the *N*-terminal cysteine residue on the protein, possibly representing intermediates during Fe(CN)_2_CO group biosynthesis. Together, these findings suggest that the HybG chaperone is involved in both biosynthesis and delivery of the Fe(CN)_2_CO group to its target protein. HybG is thus suggested to shuttle between the assembly complex and the apo-catalytic subunit. This study provides new insights into our understanding of how organometallic cofactor components are assembled on a scaffold complex and transferred to their client proteins.

## Introduction

[NiFe]-hydrogenases activate dihydrogen to release electrons and protons, or they reduce protons to help balance the redox state of bacterial and archaeal cells^1^. These enzymes harbour a bimetallic NiFe(CN)_2_CO cofactor in their catalytic subunit^2^. The Fe^2+^ ion of this cofactor is unusual because it carries a carbonyl and two cyano (CN^−^) ligands^3^, which aid catalysis^4,5^. It is still unclear how the Fe(CN)_2_CO group, which is synthesised on a distinct scaffold complex, is ultimately transferred into the apo-form of the immature catalytic subunit. Here, we use the hydrogen-oxidising hydrogenase 2 of the bacterium *Escherichia coli*^6^ as a model system to address how the Fe(CN)_2_CO group of this[NiFe]-cofactor might be transferred into the immature (apo-form; called pre-HybC) of the hydrogenase 2 catalytic subunit.

**Figure 1:**
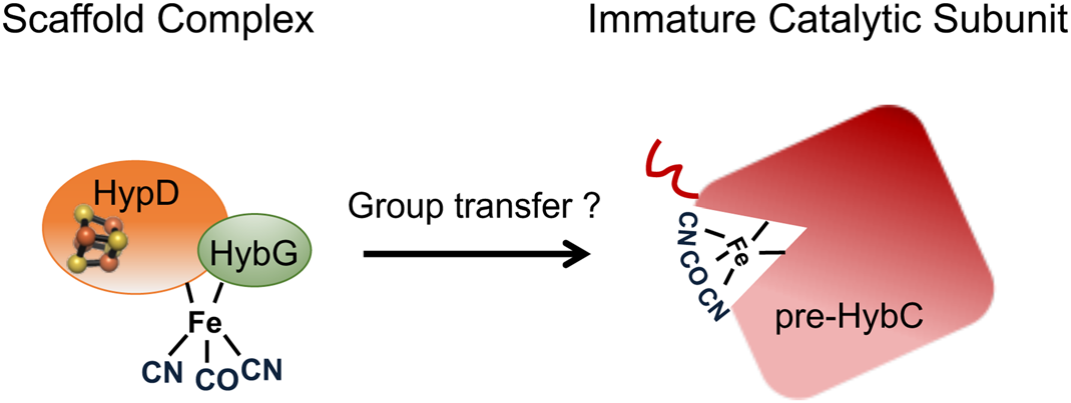
Schematic overview of iron group transfer into the apo-catalytic subunit of hydrogenase 2. The 4Fe-4S cluster in HypD is depicted as a distorted cube in HypD, while the red ‘squiggle’ on pre-HybC signifies a *C*-terminal peptide that is removed by endoproteolytic cleavage after Ni^2+^ insertion has been completed^8^.

During hydrogenase 2 assembly, two highly conserved accessory proteins, called HybG and HypD, form a scaffold complex upon which the Fe(CN)_2_CO group is synthesised before it is introduced into pre-HybC^7,8^ (Fig. 1). While HypD is an iron-sulfur (FeS) cluster-containing enzyme^9^, HybG belongs to the HypC-family of small chaperone-like proteins^7,10^. HybG and HypD each has a highly conserved cysteinyl residue, and, together, these two residues are proposed to coordinate the Fe(CN)_2_CO group on the scaffold complex. These residues are Cys41 on HypD (*E. coli* numbering) and the *N*-terminal Cys2 on HypC (and HybG)^8,11^. Conveniently, the HybG–HypD scaffold complex carrying the Fe(CN)_2_CO group can be isolated under anoxic conditions, which has allowed its spectroscopic characterization^12,13^.

Two pieces of circumstantial evidence suggest that HybG might be involved in the ultimate transfer of the Fe(CN)_2_CO group from the scaffold complex to pre-HybC. The first is based on the fact that many bacteria and archaea that synthesise more than one[NiFe]-hydrogenase often have HybG paralogues^7,14^. For example, *E. coli* has the HybG paralogue HypC, whereby these proteins share 57% amino acid sequence identity; HypC is specifically required for maturation of the catalytic subunit of the H_2_-producing hydrogenase 3^7,15^. The second piece of evidence in support of a transfer function of HypC/HybG was provided when hydrogenase apo-catalytic subunits were shown to co-purify with HypC-family proteins^16,17^. Notably, however, all attempts so far to isolate HybG, or other members of the HypC-family of proteins, with an attached Fe(CN)_2_CO group have proved unsuccessful. Surprisingly, it has, however, been possible to isolate over-produced HypD carrying the Fe(CN)_2_CO group^13^. This is possibly due to the larger HypD protein providing a more stable coordination sphere for the chemically sensitive group. Nevertheless, HybG is still essential for synthesis of the Fe(CN)_2_CO group attached to over-produced HypD^7,13^. Hence, there is still uncertainty as to which scaffold protein carries and delivers the Fe(CN)_2_CO group to the hydrogenase large subunit precursor (Fig. 1). Therefore, the aim of this study is to understand better the pathway of cofactor assembly into pre-HybC, the catalytic subunit of hydrogenase 2.

So far it has not been possible to demonstrate that HybG (or paralogues thereof) carries the Fe(CN)_2_CO group and this is likely due to the lability of this putative intermediate on the ~ 10 kDa HybG protein. Therefore, we decided to adopt a mass spectrometry approach to address this issue. Native mass spectrometry (MS) has proven to be a powerful technique that can yield unequivocal and detailed molecular information on protein modifications, and protein–protein interactions within larger protein complexes. Moreover, it can provide snapshots of biosynthetic processes, in particular allowing detection of transient intermediates^18–21^. Therefore, dynamic assemblies of proteins that are not amenable to high-resolution structural or spectroscopic techniques can be investigated by native MS^22^. In the current study, we resolve the protein interaction network between HypD, HybG and pre-HybC, the hydrogenase 2 large-subunit precursor of *E. coli*, and demonstrate that HybG indeed carries the Fe(CN)_2_CO group. The results obtained herein serve as a blueprint for the analysis of cofactor biosynthesis, and maturation of metalloproteins in general.

## Results

### Stoichiometric HybG–HypD complexes identified by native MS

**Figure 2:**
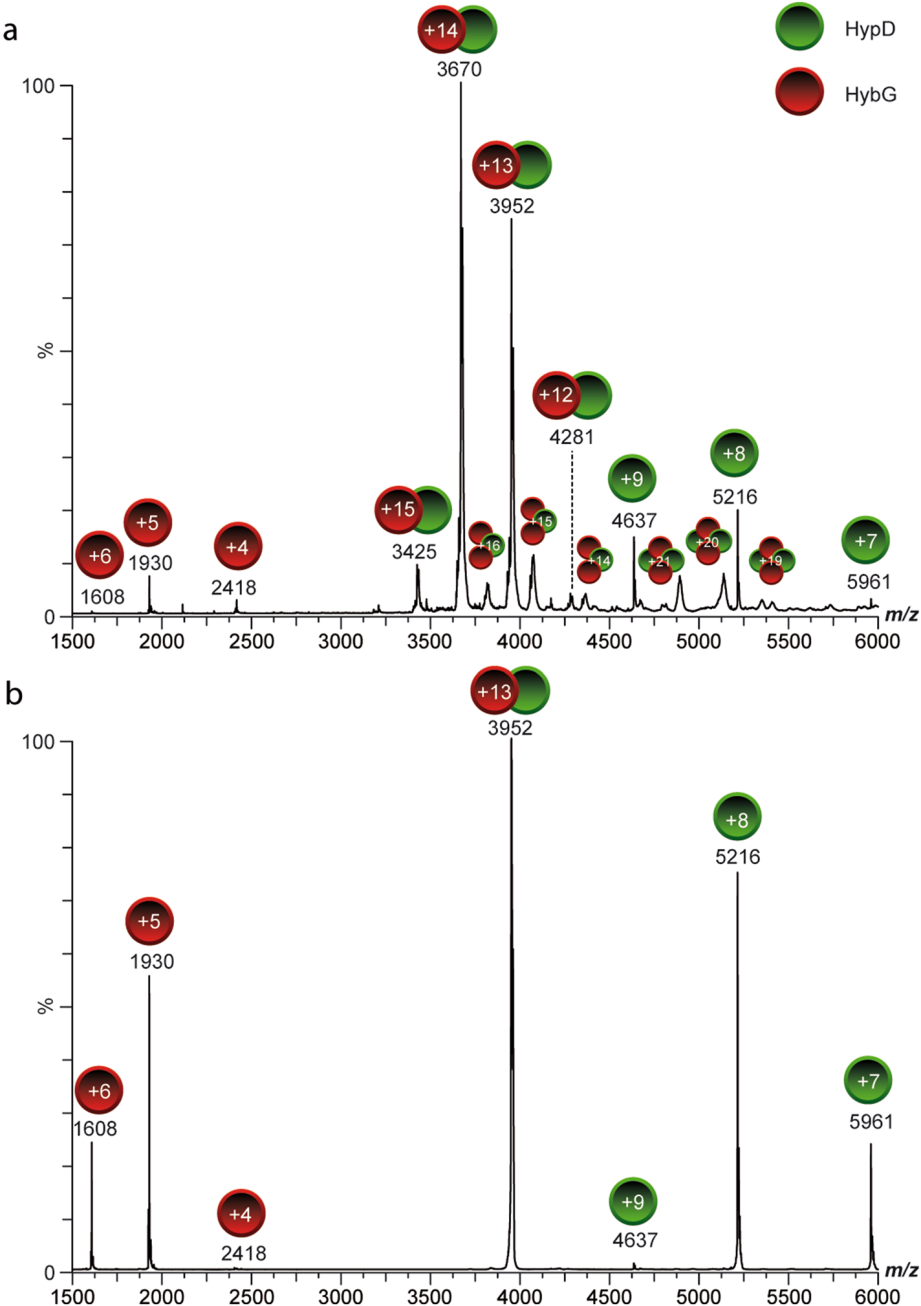
Native mass spectra of StrepII-HybG–HypD complex. Native mass spectrum of (**a**) the StrepII-HybG–HypD complex at a collision energy of 40 V. Stoichiometry of the main complex species is StrepII-HybG:HypD 1:1, with minor species showing 2:1 and 2:2 ratios (indicated by green circles for HybG and red circles for HypD). (**b**) Mass spectrum of the dissociation of the 13+-charged ion species of the heterodimer into HybG (charge states + 4 to + 6, red circles) and HypD (charge states + 7 to + 9, green circles) at a collision energy of 80 V. The corresponding measured masses of the StrepII-HybG and HypD proteins are shown in Tables S1 and S2.

The HybG–HypD complex used in this study was affinity-purified under anoxic conditions by taking advantage of the *C*-terminal StrepII-tag attached to HybG and will be referred to as HybG–HypD throughout this study; unless specifically stated otherwise, HypD was untagged. Analysis by native MS revealed a 1:1 stoichiometry for the HybG–HypD complex (Fig. 2a; see Tables S1 and S2 for measured masses). As well as minor signals arising from free HybG and HypD, which arose due to the comparatively high collision energy used in the MS experiment, minor amounts of HybG–HypD complexes with stoichiometries of 2:1 and 2:2 were also measured (Fig. 2a).

Signals for monomeric HybG and HypD were generated by dissociating the 1:1 complex using tandem MS/MS experiments (collision-induced dissociation, CID-MS/MS) of the isolated + 13 charged species of the observed complex signal (Fig. 2b). This dissociation yielded signals for highly charged HybG species (red circles, charge states + 4 to + 6, Fig. 2b) and signals corresponding to HypD (green circles, charge states + 7 to + 9, Fig. 2b) identical to those observed in the full mass spectrum (Fig. 2a).

**Figure 3:**
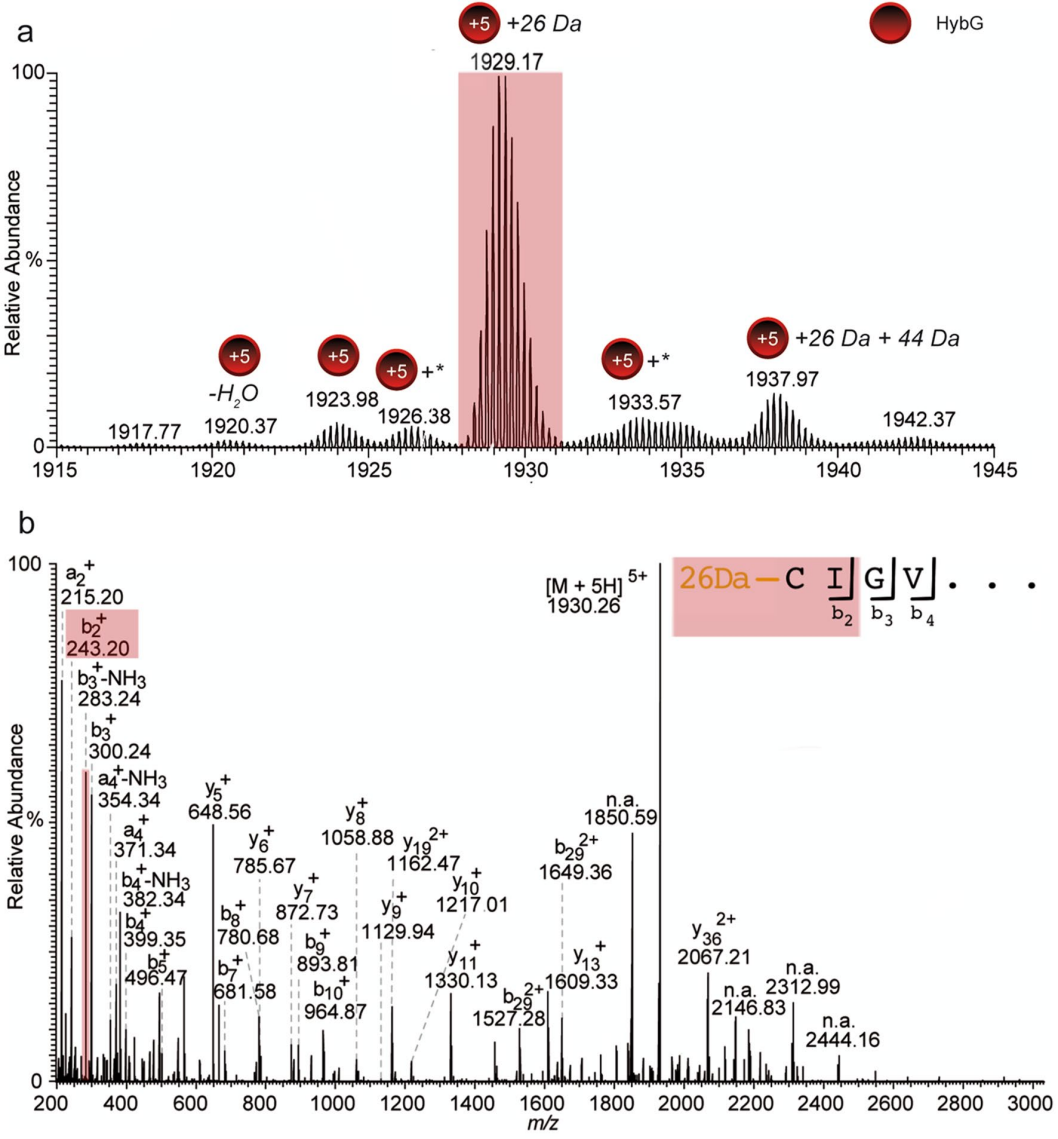
High-resolution mass spectrum of StrepII-HybG modifications. (**a**) The isotope pattern shows high-resolution StrepII-tagged HybG (5+ charge state, red circle) relevant adduct species. The asterisk (*) indicates that the modification was not clearly identifiable. (**b**) High-resolution mass spectrum of StrepII-tagged HybG with the indicated species that was isolated to generate the top-down CID fragment ion spectrum. The signature fragment ion b_2_^+^ unambiguously shows that the additional mass of 26 Da is mostly located on the *N-*terminal cysteinyl residue (see also Table S2).

StrepII-tagged HybG, when dissociated from the complex, revealed additional signals accounting for potential covalent modifications. To identify these modifications, high-resolution MS data for StrepII-tagged HybG and for StrepII-tagged HypD, purified individually, were acquired. While StrepII-tagged HypD purified in the absence of HybG was not observable in the high-resolution mass spectra, due its high susceptibility to aggregation and/or poor ionization behavior, StrepII-tagged HybG yielded the same pattern of modified ion species in comparison to the dissociation experiments of the HybG–HypD complex preparation (Fig. 3a).

High-resolution MS measurements for StrepII-tagged HybG revealed a signal accounting for the non-modified protein at *m/z* 1923.98 (+ 5 charge state, deconvoluted mass: 9615 Da), as well as the signal for a highly abundant species at *m/z* 1929.17 (+ 5 charge state, deconvoluted mass: 9641 Da; Table S2) with an additional mass of 26 Da. The latter could indicate an addition of CO_2_ (+ 44 Da) followed by a loss of a water molecule (− 18 Da). A further species at *m/z* 1937.97, corresponding to StrepII-tagged HybG with an additional mass of 70 Da, was also identified. This could correspond to the addition of another CO_2_ molecule (44 Da) and the 26 Da modification. Notably, however, in all experiments, the + 26 Da modification on HybG was present, and almost always exhibited the peak with the highest relative intensity for all HybG species. Additional weak signals were identified in the range of m/z 1933.57 ± 3, but were not clearly resolved.

As is the case for all HypC-family members, the *N-*terminal cysteinyl residue (formally amino acid position 2) of HybG is essential for the biological function of the protein^7,15,17^. To determine whether the observed modifications were dependent on this cysteinyl residue, we analyzed a mutant StrepII-tagged HybG variant in which this cysteinyl residue was substituted by an alanyl residue (StrepII-HybG_C2A_; C2A variant). By comparing high-resolution mass spectra of the native HybG with the HybG_C2A_ variant, it was observed that all of the additional modifications identified for the native HybG protein were no longer present on the HybG_C2A_ variant (Fig. S1).

Finally, to prove that the modification accounting for the mass addition of 26 Da was localized on the *N*-terminal cysteinyl residue of native StrepII-tagged HybG, we performed top-down tandem mass spectrometric experiments (CID-MS/MS) of the ion species at *m/z* 1929.17 (5+ charged, deconvoluted mass: 9641 Da, Fig. 3a). The resulting fragment ion spectrum contained a b-type ion series (b_9_ to b_2_), which clearly demonstrated that the initial cysteinyl residue was modified, and that it carried the additional mass of 26 Da (Fig. 3b).

### HybG interacts specifically with the large subunit precursor pre-HybC

**Figure 4:**
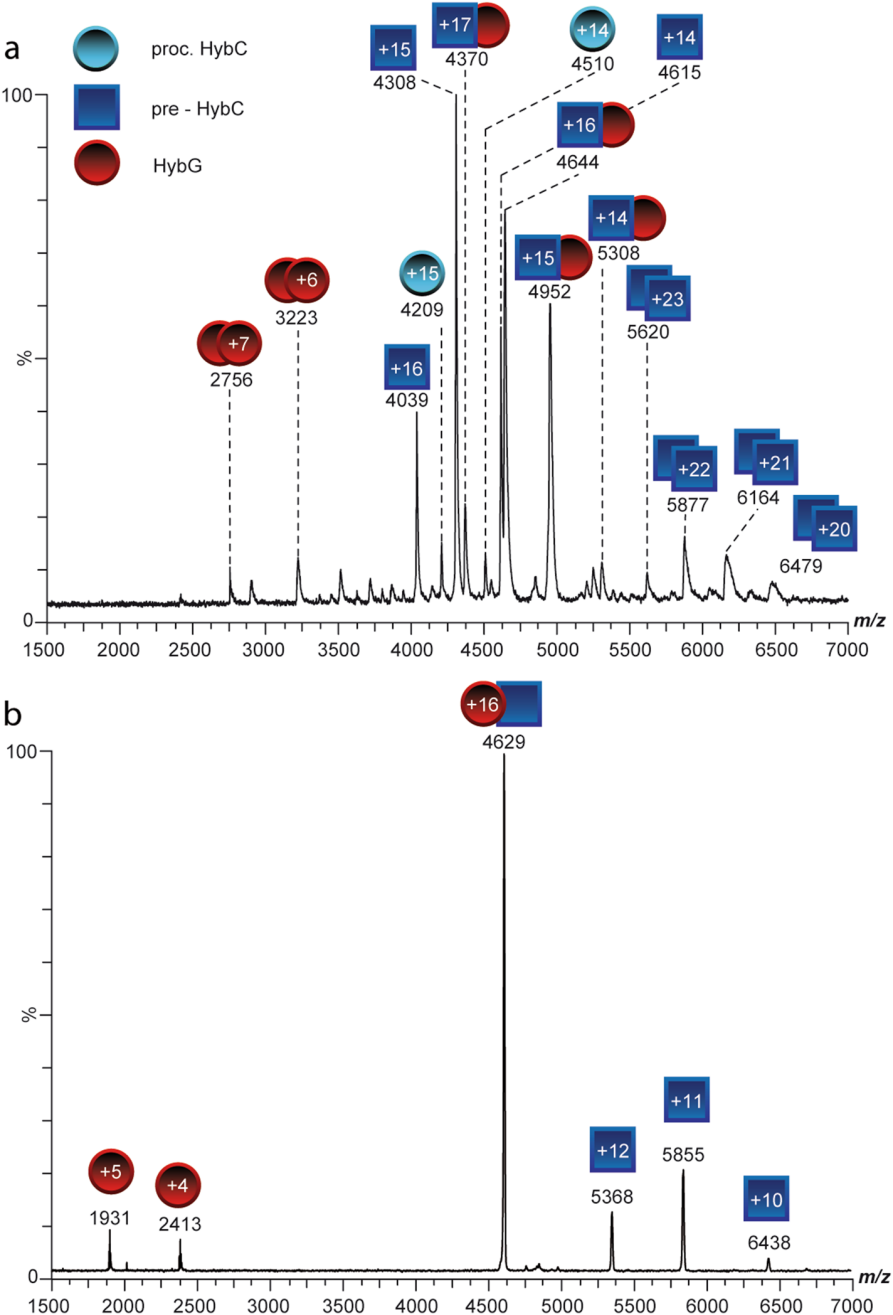
Native mass spectra of the StrepII-HybG-pre-HybC complex. (**a**) Native mass spectrum of the HybG (red circles)-pre-HybC (blue squares) heterodimeric complex. HybG has a *C-*terminal StrepII-tag and pre-HybC carries a *N*-terminal His-tag. *C*-terminally truncated HybC (light blue circles) and pre-HybC dimers, as well as minor amounts of HybG dimers were also observed. (**b**) The 16+ charged complex species was isolated and dissociated by collision-induced dissociation (CID) resulting in HybG monomer (+ 4 and + 5; red circles) and pre-HybC monomer (+ 10 to + 12, blue squares). The corresponding measured masses of pre-HybC and mature HybC are shown in Table S1.

The apo-form of the hydrogenase 2 large subunit, pre-HybC, is a target for the delivery of the Fe(CN)_2_CO group of the cofactor after its synthesis has been completed on the HybG–HypD scaffold complex^6,8,23,24^. Pre-HybC is subsequently converted to its mature species^25,26^, which we refer to here as HybC. It is unresolved whether transfer of Fe(CN)_2_CO to pre-HybC is mediated by the HybG–HypD scaffold, or HybG, or HypD. Therefore, to address this, we first tested whether the interaction between HybG with pre-HybC could be resolved using native MS. During analysis of a purified, *N-*terminally His-tagged pre-HybC preparation from *E. coli,* we detected an ion species corresponding to the expected mass of pre-HybC (Fig. 4a, dark blue squares; see also Table S1). Strikingly, we also detected minor amounts of processed HybC (Fig. 4a, light blue circles; see also Table S1), the origin of which remains unresolved. Purified *N*-terminally His-tagged pre-HybC was then supplemented with equimolar amounts of HybG to identify any complex formation between the proteins. Upon addition of HybG, signals were immediately observed that indicated the formation of a 1:1 HybG/pre-HybC heterodimeric complex (Fig. 4a, red circle + dark blue square). Notably, small amounts of HybG dimers were also observed, but these were not detected in complexes with pre-HybC and may result from the tendency of HybG to aggregate when not in complex with one of its partner proteins (see Fig. S2;^27^). The identity of the HybG/pre-HybC complex was again confirmed by tandem MS experiments (CID-MS/MS), whereby the + 16 charged ion species of the complex was dissociated into monomeric HybG (red circles) and monomeric pre-HybC (dark blue squares, Fig. 4b) species. Interestingly, we were never able to detect a complex between HybG and mature HybC, which is in agreement with previous observations^15,17,25,26^.

Using pull-down interaction studies, it was also shown that when StrepII-tagged HybG was incubated with His-tagged pre-HybC, and the mixture was applied to a StrepTactin column, pre-HybC co-eluted with HybG (Fig. S2a). When this experiment was performed with the mutant HybG_C2A_ variant, no retention of pre-HybC on the column was observed (Fig. S2b). This result indicates that the *N-*terminal cysteinyl residue of HybG is essential for the interaction with pre-HybC to occur.

### Pre-HybC titrates HybG from the HybG–HypD complex

**Figure 5:**
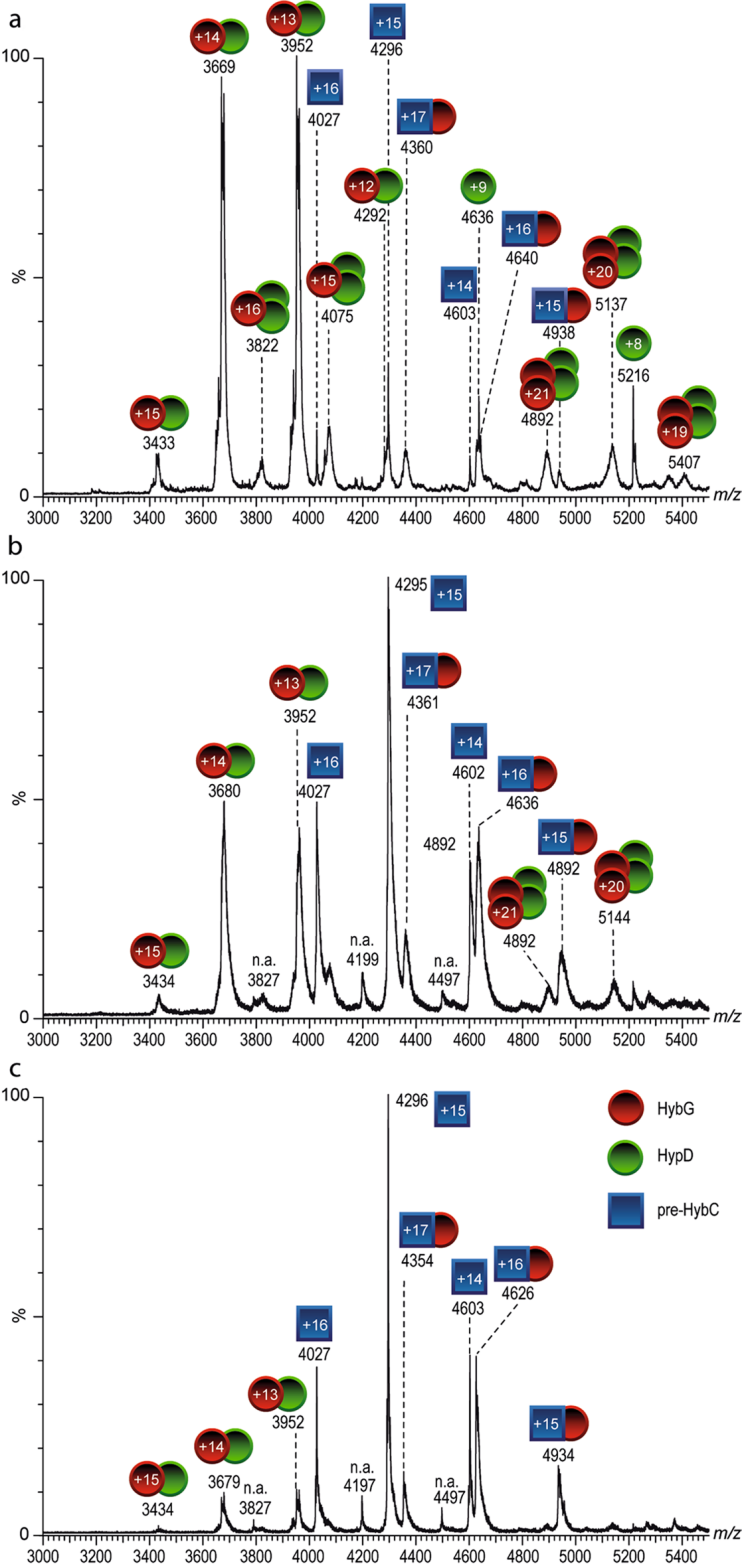
Native mass spectra showing pre-HybC titrating HybG from the HybG–HypD complex. Native mass spectrum of the StrepII-tagged HybG (red circles)-HypD (green circles) complex upon addition of increasing amounts of *N*-terminally His-tagged pre-HybC (blue squares). Ratios of pre-HybC to HybG–HypD complex were (**a**) 1:20, (**b**) 1:6, (**c**) 1:3.

The data obtained so far clearly indicate that HybG interacts specifically with pre-HybC. To analyse whether pre-HybC also can interact with the HybG–HypD scaffold complex, increasing concentrations of His-tagged pre-HybC were added to the HybG–HypD complex (Fig. 5; molar ratios of pre-HybC to HypD–HybG complex ranged from 1:20 up to 1:3). The formation of a HybG-pre-HybC complex was observed directly upon addition of a low concentration of His-tagged pre-HybC (Fig. 5a), whereby StrepII-HybG could only have originated from the HybG–HypD complex. Increasing the concentration of His-tagged pre-HybC caused a concomitant increase in the signal intensity of the HybG-pre-HybC complex, while the intensity of the HybG–HypD complex decreased correspondingly (Fig. 5b,c). Pre-HybC was thus able to titrate HybG from the HybG–HypD complex, even at a molar ratio of ~ 1:20 in favour of HybG–HypD (Fig. 5a). The removal of HybG from the HybG–HypD complex appeared to have a negative impact on the stability of HypD, and probably led to its aggregation. Therefore, non-complexed HypD was not detected in the native mass spectra at higher concentrations of pre-HybC, which correlates with our earlier findings (see Fig. 2). Importantly, HypD was never identified to interact with pre-HybC, or to enter into a ternary complex with pre-HybC and HybG (Fig. 5). This indicates that HybG probably delivers the Fe(CN)_2_CO group to pre-HybC.

### StrepII-tagged HybG carries the Fe(CN)_2_CO group

**Figure 6:**
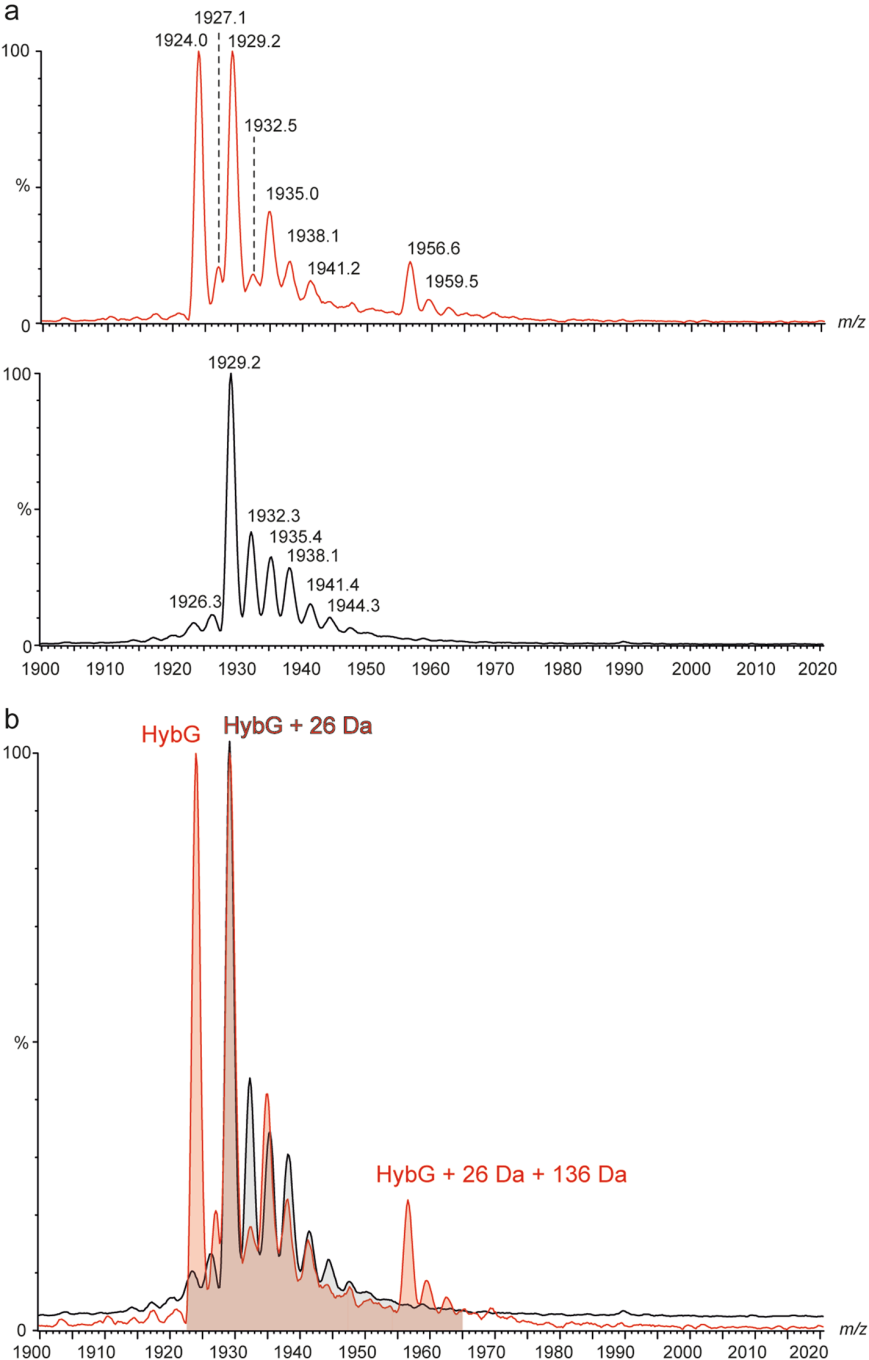
Native mass spectrum of HybG carrying the Fe(CN)_2_CO group after dissociation from the HybG–HypD complex. (**a**) StrepII-HybG–HypD (upper panel) or StrepII-HybG–HypD_C41A_ (lower panel) complexes were purified anaerobically after overproduction in *E. coli* DHP-D. Both StrepII-HybG spectra were generated by dissociation in MS mode without isolating a precursor ion. Collision energy was set to 80 V. (**b**) Overlay of both HybG spectra shown in part (**a**). Note that while both spectra appear to include a mixture of minor oxidation products, some minor modifications were only observed on the HybG from the native complex. These minor species were not identified.

In an attempt to identify a HybG species carrying the Fe(CN)_2_CO group, while simultaneously minimising detrimental exposure of the complex to oxygen, we performed native MS experiments on anaerobically isolated HybG–HypD complex after rapid buffer-exchange^28^. We also isolated a StrepII-HybG–HypD_C41A_ complex in which native StrepII-tagged HybG was in complex with a mutant HypD_C41A_ protein^29^. The exchange of C41 for an alanyl residue inactivates HypD and prevents synthesis of the Fe(CN)_2_CO group^9^. Both complexes (HybG–HypD and HybG–HypD_C41A_) were analysed by native MS, whereby we focused on the m/z region in the spectra harbouring the signals for dissociated HybG. Surprisingly, both spectra differed substantially from one another, despite the fact that both measured the same dissociated, native StrepII-tagged HybG protein (Fig. 6a). The mass spectrum of HybG dissociated from the native HybG–HypD complex revealed two main ion species. One ion species had a m/z 1929.2 (+ 5 charge state, deconvoluted mass: 9641 Da; Table S2), and one had an equally intense species at m/z 1924 (+ 5 charge state, deconvoluted mass: 9615 Da), which indicated the presence of a population of StrepII-HybG without any modification. The unique feature of this spectrum, however, was the presence of a signal at m/z 1956.6 (+ 5 charge state, deconvoluted mass: 9783 Da; Table S2), which showed StrepII-HybG modified with 26 Da plus an additional 136 Da, which is the mass of the Fe(CN)_2_CO group (Fig. 6a, upper panel). In contrast, StrepII-HybG that dissociated from the complex with HypD_C41A_ showed a main ion species at m/z 1929.2 indicating HybG with the + 26 Da modification, but no unmodified HybG species. Importantly, however, no signal at m/z 1956.6 was observed (Fig. 6a lower panel), which is consistent with the inability of HypD_C41A_ to synthesise the Fe(CN)_2_CO group. Furthermore, we observed minor species showing successive mass increases of ~ 16 Da, suggesting successive oxidation events that may have occurred either in solution or during the ESI process (Fig. 6a, lower panel). An overlay of both spectra highlights that only the StrepII-HybG protein dissociated from the native HypD scaffold complex carries the Fe(CN)_2_CO group (Fig. 6b).

## Discussion

The findings presented in this study show that HybG, a member of the large and conserved HypC family of [NiFe]-hydrogenase accessory maturation proteins, shuttles between the HypD scaffold protein and its cognate hydrogenase 2 large subunit precursor, pre-HybC. HybG is thus not only involved in the biosynthesis of the Fe(CN)_2_CO group, but also is proposed to transfer it to pre-HybC. Although we could not demonstrate the direct transfer of Fe(CN)_2_CO from HybG to pre-HybC in the current study, we show unambiguously that HybG enters a 1:1 complex with pre-HybC, and that it carries the Fe(CN)_2_CO group. HypD, in contrast, does not enter into a ternary complex with HybG and pre-HybC. This indicates that HypD acts solely as a component of the central scaffold, where it serves to synthesise the Fe(CN)_2_CO group^12,13^.

**Figure 7:**
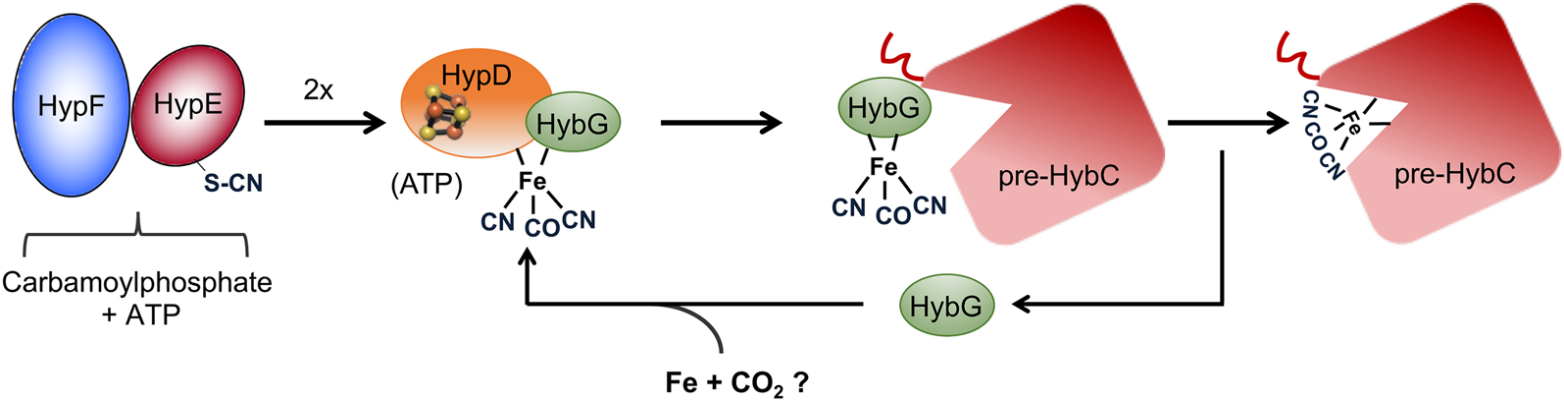
Model depicting the interaction network of HybG in [NiFe]-hydrogenase 2 large subunit maturation (see text for details). The small ball-and-stick representation within HypD indicates a 4Fe-4S cluster. The *C-*terminally located thiocyanate on HypE is represented by –S–CN^31^; the penta-coordinated Fe ion attached to the HybG–HypD complex depicts the Fe(CN)_2_CO group. Pre-HybC, the apo-form of the catalytic subunit of hydrogenase 2.

There is strong evidence indicating that the cysteinyl residue at position 41 in HypD coordinates the Fe(CN)_2_CO group^13,30^. When we dissociated native HybG from an anaerobically isolated scaffold complex in which C41 on HypD had been exchanged for an alanyl residue, HybG lacked bound Fe(CN)_2_CO. This experimental evidence strongly supports the contention that the iron group is coordinated via C41 on HypD. Moreover, exchange of HybG’s *N*-terminal cysteinyl residue for an alanyl residue is known to prevent synthesis of active hydrogenase 2 in vivo^30^. This also supports the contention that C2 of HybG is the other main coordination site for the Fe(CN)_2_CO group on the scaffold complex^8^. Finally, we show here that the *N*-terminal cysteine on HybG is also required for interaction with pre-HybC (see model in Fig. 7), which indirectly supports a role for this residue in the delivery of the Fe(CN)_2_CO group to the active site cavity of pre-HybC^15^. The demonstration of direct transfer of the Fe(CN)_2_CO group from HybG to pre-HybC, presumably via a thiol-exchange reaction^8^, could not be achieved in the current study and will be elucidated further in future work.

It was notable that HybG with the bound Fe(CN)_2_CO group also had a further 26 Da modification directly associated with the *N*-terminal cysteinyl residue. The same 26 Da modification was also observed on HypC and on HybG isolated from a mutant strain lacking HypD (data not shown), which indicates that its provenance is independent of the hydrogenase accessory protein machinery. One explanation for this 26 Da mass increase is that it might arise through initial addition of CO_2_ (44 Da), resulting from an anaerobic decarboxylase reaction, followed by the loss of water (18 Da). Cyanide transfer via a HypE-dependent thiol-transferase side-reaction was also considered as an alternative source of this modification. The only way cyanide might yield a + 26 Da addition is, however, if one invokes generation of a transient radical species, to which a proton is then added. Consequently, this possibility is currently considered to be unlikely. Future analyses will be, however, required to define precisely the (bio)chemical origin of the modification and whether it is on the biosynthetic pathway of the Fe(CN)_2_CO group in vivo.

In addition to the 26 Da modification, the other minor modification to HybG identified was a further mass shift of 44 Da, attributable to binding of CO_2_. Modification by addition of CO_2_ has been identified previously using infrared spectroscopy and proposed as a potential source of the diatomic CO ligand^27^. Notably, this 44 Da modification also depends on the *N*-terminal cysteinyl residue, because conversion to an alanyl residue in the HybG_C2A_ variant resulted in loss of all modifications of HybG. Moreover, the 44 Da modification was not observed on the species of HybG carrying the Fe(CN)_2_CO group. This suggests that at least two populations of HybG exist: one species that has the 44 Da (+ CO_2_) modification, while the other carries the Fe(CN)_2_CO group. Notably, however, both species also have the 26 Da (+ CO_2_ –H_2_O) modification on the cysteine at the *N*-terminus.

As well as interacting with the apo-form of the hydrogenase catalytic subunit, HybG (and other HypC-family proteins) also has an essential role in biosynthesis of the Fe(CN)_2_CO metal center. Data from a recent study suggest that HybG might even function in reductive synthesis of the CO and CN^−^ ligands on the iron^29^. It might also be involved in recruiting the iron and CO_2_ molecules for further modification by HypD^24,27^ (see model in Fig. 7). These features of HybG are reminiscent of the role of the IscA/SufA carrier protein family in iron-sulfur cluster assembly^32–34^. In the case of hydrogenase maturation, the HybG–HypD complex is supplied with CN^−^ ligands, originating from carbamoylphosphate^35^, and these ligands are generated and provided by two further accessory proteins HypE and HypF^31,36^. This modular form of cluster biosynthesis possibly represents an archetype for many complex metal-based cofactors that use a separate scaffold complex for cofactor synthesis, including, for example, molybdenum-iron cofactor biosynthesis for nitrogenase^37^. In the case of hydrogenase maturation, the controlled delivery of components to, and transfer from, the central HybG–HypD scaffold (Fig. 7) allows introduction of both temporal and regulatory checkpoints that help ensure correct and efficient cofactor synthesis, and ultimately enzyme assembly.

Based on the findings of the current study, the use of native MS to examine [NiFe]-cofactor biogenesis adds a new analytical dimension to dissect this maturation process. The results obtained in this study suggest that this approach can be used to investigate the temporal order of complex formation from the initiation of cofactor assembly to its ultimate insertion into the target apo-catalytic subunit. Based on the evidence presented here, this approach also allows identification of transient, unstable intermediates. Native MS also provides information on the stoichiometry of the protein complexes involved, and can even be used to determine changes in the redox states of metal clusters^18^. Future native MS studies on the hydrogenase maturation system will focus on optimising preparative steps so that transfer of Fe(CN)_2_CO between HybG and pre-HybC can be examined in detail.

## Materials and methods

### Bacterial strains, plasmids and growth conditions

The *E. coli* strains used included MC4100 (F^−^, *araD139,* ∆(*argF-lac*)*U169,* λ^−^, *rpsL150, relA1*, *deoC1, flhD5301*, ∆(*fruK-yeiR*)*725*(*fruA25*), *rbsR22*, ∆(*fimB-fimE*)^38^, its isogenic mutant derivatives BEF314 (like MC4100, but Δ*hypB-E*)^39^, and DHP-D (Δ*hypD*)^39^ and BL21(DE3) (F^−^ *ompT gal dcm lon hsdS*_B_(*r*_*B*_^*−*^* m*_*B*_^*−*^*)* λ(DE3 [*lacI lacUV5*-T7 *gene 1 ind1 sam7 nin5*]) (Novagen, USA). The plasmids used included pASK-hybG, pASKhybG_C2A_^30^, pT-hybG-hypDEF^40^, pT-hybG-hypD_C41A_EF^28^, and pCAN-hybC_stop_^41^. pCAN-hybC_proc_ was created by introducing an ochre stop at codon position 553 in the *hybC* gene on plasmid pCAN-hybC_stop_ using site-directed mutagenesis (QuikChange II mutagenesis kit, Agilent) employing the oligonucleotides HybC_fwd_ (5′-GCCTGTGCGGTACACTAAGTGGATGCTGACGGC-3′) and HybC_rev_ (5′-GCCGTCAGCATCCACTTAGTGTACCGCACAGGC-3′).

*E. coli* strain BL21(DE3) was used as host for general overproduction of native StrepII-HybG–HypD complexes, while BEF314 (Δ*hypB-E*) or DHP-D (Δ*hypD*) was used to overproduce native StrepII-HybG–HypD and StrepII-HybG–HypD_C41A_ protein complexes for analysis of HybG modifications. *E. coli* strains were transformed with the indicated plasmid using standard procedures^42^ and cultivated in modified TB medium^43^. Depending on the plasmid used, the medium contained either 100 μg ml^−1^ of ampicillin or 15 μg ml^−1^ of chloramphenicol to maintain plasmid selection. Cultivation was at 30 °C on a rotary shaker and incubation was continued until an optical density at 600 nm of 0.4 was reached. Expression of plasmid-borne *hybG*, or *hybG* plus the *hyp* genes, and which were under the control of the *araBAD* promoter, was induced by the addition of 0.2 μg ml^−1^ anhydrotetracycline (AHT) followed by continuing incubation at 30 °C for a further 3–5 h. Expression of the *hybC* gene variants on plasmids was induced by the addition of 0.1 mM isopropyl β-D-1-thiogalactopyranoside (IPTG) followed by further incubation of the culture at 30 °C for 3–5 h. Cells were harvested (OD_600nm_ of between 1.0 and 1.2) by centrifugation of the culture for 15 min at 50,000 g at 4 °C and washed cell pellets were either used immediately or stored at − 20 °C until use.

### Protein purification

All steps in cell disruption and protein purification were carried out under anoxic conditions in an anaerobic chamber (Coy Laboratories, Grass Lake, USA). Cells were suspended in 2 ml of 50 mM Tris–HCl, pH 8 containing 150 mM NaCl g^−1^ wet weight of cell paste. PMSF (phenylmethylsulfonyl fluoride) was added to a final concentration 0.8 mM and DNAse I was added to 10 μg ml^−1^ to the cell suspension and cells were disrupted by sonication (Sonotrode, 20–40 W with 0.5 s pulses for 5 min) on ice. Unbroken cells and cell debris were removed by centrifugation of the suspension at 45,000×*g* for 20 min at 4 °C. The resulting crude extract was used directly for protein purification.

The StrepII-tagged HybG derivatives or the HybG–HypD complexes were purified using StrepTactin Sepharose® (IBA, Göttingen) exactly as described^27^. *N*-terminally His-tagged pre-HybC and HybC_proc_ were purified using cobalt-charged TALON® Superflow™ agarose (Cytiva) exactly as described^41^. When required, eluted, pooled protein fractions from the affinity chromatography steps were immediately buffer-exchanged into anaerobic 50 mM Tris–HCl, pH 8, using 5 ml PD-10 columns containing G-25 Sephadex matrix (Cytiva) and then chromatographed on a 1 ml Q-Sepharose® fast-flow column (Cytiva) equilibrated with the same buffer. Bound proteins were eluted stepwise using equilibration buffer containing 50, 150, 300 and 500 mM NaCl. Subsequently, samples were again buffer-exchanged into anaerobic 50 mM Tris–HCl, pH 8, containing 150 mM NaCl (buffer A). Protein samples were concentrated using Amicon centrifugal concentration filters (cut-off of 5, 10, 30, or 50 kDa, depending on the protein sample) and samples were stored at − 80 °C.

Protein concentration was determined as described^44^.

### Pull-down assays

All experiments performed in this study used StrepII-tagged HybG (*C-*terminal tag), a *N*-terminally His-tagged pre-HybC or HybC_proc_, and HypD that was untagged. To examine the interaction between StrepII-tagged HybG or HypD–HybG with His-tagged pre-HybC or HybC_proc_, 150 μg (15 nmole) of each protein, or protein complex, were mixed and incubated for 30 min at 30 °C. After incubation, the mixture was loaded onto either a 0.5 ml StrepTactin Sepharose or a 0.5 ml nickel-NTA (Qiagen) column for the purification of StrepII- or His-tagged complexes, respectively. Each column was equilibrated with buffer A (see above). After sample-loading, columns were washed with 10 column volumes of buffer A and bound proteins were eluted from the StrepTactin column with buffer A containing 50 mM biotin, or from the Ni–NTA column with buffer A containing 300 mM imidazole. Elution fractions (E) of 0.5 ml were collected and analyzed immediately by electrophoresis on 12.5% (w/v) acrylamide SDS-PAGE^45^. Gels were subsequently stained with silver (Pierce™ Silver-staining Kit, Thermo Fischer), or were transferred to nitrocellulose membranes and challenged with antiserum (diluted 1:1000) raised against purified HybG.

### Mass spectrometry analyses

For native MS measurements, as well as high resolution MS measurements, the buffer was exchanged to 500 mM ammonium acetate, pH 6.8, by using Amicon Ultra centrifugal filter units with an appropriate molecular weight cut-off (Merck Millipore, Darmstadt), unless stated otherwise.

The concentration of protein solutions after buffer-exchange was approximately 10 μM. Native MS was carried out on a High-Mass Q-TOF II instrument (Waters Micromass/MS Vision) equipped with a nano-electrospray ionization (ESI) source. The applied capillary voltage ranged from 1.0 to 1.4 kV, while the sample cone voltage varied from 100 to 160 V. The source pressure was adjusted to 9.2–10 mbar and the pressure in the collision cell was adjusted to 10^−2^ to 210^−2^ mbar. MS measurements were carried out using MS profile mode for the quadrupole to guide ions within the *m/z* region of interest. The acceleration voltage in the collision cell was set to 30–60 V for MS measurements. Dissociation experiments were carried out by collision-induced dissociation (CID) for the selected ion species. To achieve dissociation of protein complexes the acceleration voltage of the collision cell was increased in dependency of the measured complex (ranging between 60 and 90 V). Data were recalibrated by using cesium iodide (CsI).

High-resolution ESI–MS and MS/MS data were recorded on an Orbitrap Fusion Tribrid mass spectrometer (Thermo Fisher Scientific) equipped with a nano-ESI source. The resolving power was set to 120,000 at *m/z* 200. The fragment ion mass spectra (CID-MS/MS) were obtained in the linear ion trap.

### Rapid online buffer exchange

Online buffer exchange (OBE) was carried out on an Ultimate 3000 RSLC nano-HPLC system (Thermo Fischer Scientific) using a size-exclusion column with a length of 12 cm, an inner diameter of 0.6 mm using Bio-Gel P6-Resin (Biorad) that was prepared in-house, as described in^28^. Buffer-exchange was not performed anaerobically. After equilibration of the column with target buffer solution (250 mM ammonium acetate, pH 6.8), 5 µl of 20 µM protein solution was injected onto the column. Buffer exchange was achieved by isocratic elution with a flow rate of 10 µl/min. The nano-HPLC system was directly coupled to a nano-ESI source (Waters) equipped with a stainless-steel emitter. The transfer flow of eluted protein from the SEC-column to the nano-ESI source was set to 300 nL min^−1^. The chromatographic step was tracked by UV-absorption at a wavelength of 280 nm. The method was adapted from^28^. The applied capillary voltage was adjusted to 2.0–2.3 kV for the OBE measurements, while all other settings were as described before.

## Supplementary Information

Supplementary Information.
