# Supplementary material for: Native mass spectrometry identifies the HybG chaperone as carrier of the Fe(CN)2CO group during maturation of E. coli [NiFe]-hydrogenase 2
Source: Sci Rep. 2021 Dec 21;11:24362. doi: 10.1038/s41598-021-03900-w (PMC8692609; doi:10.1038/s41598-021-03900-w)
Supplement: Supplementary file 1 — Supplementary Information. [file 41598_2021_3900_MOESM1_ESM.pdf]

Supplementary Information

Native Mass Spectrometry Identifies the HybG Chaperone as Carrier of the $Fe(CN)_2CO$ Group during Maturation of *E. coli* [NiFe]-Hydrogenase 2

Christian Arlt[1], Kerstin Nutschan[2], Alexander Haase[2], Christian Ihling[1], Dirk Tänzler[1], Andrea Sinz[1*] and R. Gary Sawers[2*]

[1]Institute of Pharmacy, Center for Structural Mass Spectrometry, Martin-Luther University Halle-Wittenberg, Kurt-Mothes-Str. 3a, 06120 Halle (Saale), Germany, [2]Institute for Biology/ Microbiology, Martin-Luther University Halle-Wittenberg, Kurt-Mothes-Str. 3, 06120 Halle (Saale), Germany

* Send correspondence to: Andrea Sinz, Institute of Pharmacy, Center for Structural Mass Spectrometry, Martin-Luther University Halle-Wittenberg, Kurt-Mothes-Str. 3a, 06120 Halle (Saale), Germany. Tel: +49 345 5525170, Email: andrea.sinz@pharmazie.uni-halle.de, Orchid: https://orcid.org/0000-0003-1521-4899

or Gary Sawers, Institute for Biology/ Microbiology, Martin-Luther University Halle-Wittenberg, Kurt-Mothes-Str. 3, 06120 Halle (Saale), Germany. Tel: +49 345 5526350; Email: gary.sawers@mikrobiologie.uni-halle.de, Orchid: https://orcid.org/0000-0003-0862-2683

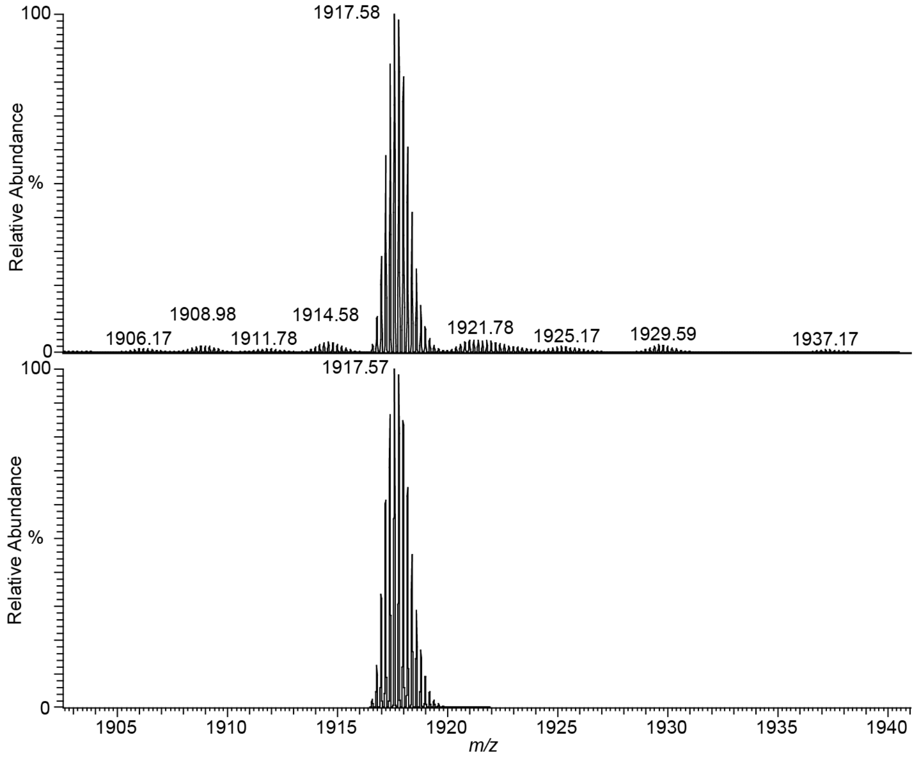

Figure S1. StrepII-HybG$_{C2A}$ variant carries no modifications. A high-resolution mass spectrum of StrepII-tagged HybG$_{C2A}$ (upper panel) and a simulated spectrum of unmodified HybG$_{C2A}$ (lower panel) are shown.

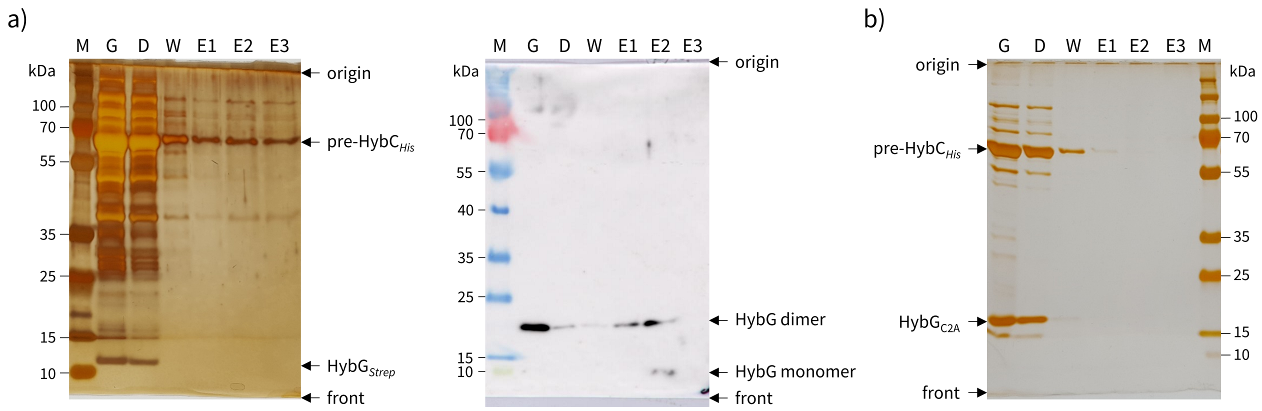

Figure S2. Pre-HybC interacts with StrepII-tagged HybG but not with the StrepII-HybG$_{C2A}$ variant. a) Aliquots of enriched extracts (150 µg) including HybG$_{Strep}$ or pre-HybC$_{His}$ were mixed and incubated for 30 min at 30 °C and then separated on a StrepTactin Sepharose column (see Materials and Methods in main text). Eluted proteins were analyzed by SDS-PAGE followed by silver-staining. Lanes: G, mixed, enriched extracts; Ft, flow-through and unbound proteins; W, wash fraction; E1-E3, aliquots of elution fractions. Note HybG strains poorly with silver. B. Western blot of the gel shown in A challenged with anti-HybG antiserum. C. The same experiment as shown in A, but performed with aliquots of purified *N*-terminally His-tagged pre-HybC and StrepII-HybG$_{C2A}$ variant. All three gels show the complete gel, including markers, from the top of the separating gel (labeled origin) to the dye-front (labeled front). The silver-stained gels were photographed directly after stained and only the contrast was adjusted using Adobe-Photoshop prior to generation of a final tif file. Signals on the western blot were detected with the Immunodetection kit SuperSignal West Pico PLUS (Thermo Scientific) and the imager Amersham Imager 600 (GE Healthcare Bio-Sciences AB) was used to record and document the data.

Table S1. Summary of averaged (by relative intensity) molecular masses of the investigated proteins by native mass spectrometry.

Protein	Measured Mass [Da]	Figure number
StrepII-HybG	9655	2, 4, 5,
Pre-HybC (with His-tag)	64400	4, and 5
Mature HybC (with His-tag)	63120	4, and 5
HypD	41720	2, and 5

Table S2. Summary of HybG species observed in native mass spectrometry measurements.

Name	*m/z (z=5)*	Measured Mass [Da][a]	Figure number
StrepII-HybG	1924.0	9615.0	3, 6 (red, black)
StrepII-HybG + 11.5 Da	1926.3	9626.5	3, 6 (black)
StrepII-HybG + 15.5 Da	1927.1	9630.5	6 (red)
StrepII-HybG + 26.0 Da	1929.2	9641.0	3, 6 (red, black)
StrepII-HybG + 41.5 Da	1932.3	9656.5	6 (black)
StrepII-HybG + 42.5 Da	1932.5	9657.5	3, 6 (red)
StrepII-HybG + 55.0 Da	1935.0	9670.0	3, 6 (red)
StrepII-HybG + 57.0 Da	1935.4	9672.0	6 (black)
StrepII-HybG + 70.5 Da	1938.1	9685.5	3, 6 (red,black)
StrepII-HybG + 86.0 Da	1941.2	9701.0	6 (red)
StrepII-HybG + 87.0 Da	1941.4	9702.0	6 (black)
StrepII-HybG + 101.5 Da	1944.3	9716.5	6 (black)
StrepII-HybG + 163.0 Da	1956.6	9778.0	6 (red)
StrepII-HybG + 177.5 Da	1959.5	9792.5	6 (red)

[a] Note that mass differences cannot unambiguously be assigned to distinct modifications.
